# High-Mass Loading Hierarchically Porous Activated Carbon Electrode for Pouch-Type Supercapacitors with Propylene Carbonate-Based Electrolyte

**DOI:** 10.3390/nano11030785

**Published:** 2021-03-19

**Authors:** Tai-Feng Hung, Tzu-Hsien Hsieh, Feng-Shun Tseng, Lu-Yu Wang, Chang-Chung Yang, Chun-Chen Yang

**Affiliations:** 1 Battery Research Center of Green Energy, Ming Chi University of Technology, 84 Gungjuan Rd., Taishan Dist., New Taipei City 24301, Taiwan; 2 Green Technology Research Institute, CPC Corporation, 2 Zuonan Rd., Nan-Tsu Dist., Kaohsiung 81126, Taiwan; 295931@cpc.com.tw; 3 Energy Storage Technology Division, Green Energy & Environment Research Laboratories, Industrial Technology Research Institute, 301 Gaofa 3rd Rd., Guiren Dist., Tainan 71150, Taiwan; fengshuntseng@itri.org.tw (F.-S.T.); louisw@itri.org.tw (L.-Y.W.); ccyang@itri.org.tw (C.-C.Y.); 4 Department of Chemical Engineering, Ming Chi University of Technology, 84 Gungjuan Rd., Taishan Dist., New Taipei City 24301, Taiwan; 5 Department of Chemical and Materials Engineering, Chang Gung University, 259 Wenhua 1st Rd., Guishan Dist., Taoyuan 33302, Taiwan

**Keywords:** supercapacitors, hierarchical porous activated carbon, high-mass loading electrode, propylene carbonate-based electrolyte, capacitive performance

## Abstract

Rational design and development of the electrodes with high-mass loading yet maintaining the excellent electrochemical properties are significant for a variety of electrochemical energy storage applications. In comparison with the slurry-casted electrode, herein, a hierarchically porous activated carbon (HPAC) electrode with higher mass loading (8.3 ± 0.2 mg/cm^2^) is successfully prepared. The pouch-type symmetric device (1 cell) with the propylene carbonate-based electrolyte shows the rate capability (7.1 F at 1 mA/cm^2^ and 4.8 F at 10 mA/cm^2^) and the cycling stability (83% at 12,000 cycles). On the other hand, an initial discharge capacitance of 32.4 F and the capacitance retention of 96% after 30,000 cycles are delivered from a pouch-type symmetric supercapacitor (five cells). The corresponding electrochemical performances are attributed to the fascinating properties of the HPAC and the synergistic features of the resulting electrode.

## 1. Introduction

With the incessant development of renewable energy sources, highly stable energy storage devices are desired to smooth the intermittent electricity output, provide the frequency regulation services, improve the system reliability, etc. Among the various energy storage technologies, the electrochemical type is widely used for the purposes mentioned above because of their fast response time, no geographical restrictions, and adjustable output based on the requirements. According to the information of the global energy storage database funded by the Department of Energy (DOE) in the United States, it is noticed that about 61% of demonstration projects utilized electrochemical energy storage technology [1]. Supercapacitors (SCs) have been considered as promising energy storage devices owing to their high-power density, excellent cycling performance, and lightweight characteristic as compared with batteries and traditional capacitors [2,3,4]. Based on the advantages of SCs, it is revealed that 34 of the demonstration projects adopting the combination of batteries and SCs or only the SCs as alternative energy storage devices were recorded in the global energy storage database, showing the practical applications of SCs [1].

Hierarchical porous activated carbon (HPAC) with very high specific surface area (normally more than 1000 m^2^/g) is known for its outstanding energy storage performances as the active electrode materials in SCs that store electricity via the electrochemical double layer [5,6,7,8]. The energy storage capabilities of HPAC are primarily determined by the physical features, especially the porous structure. Specifically, the macropores (>50 nm) serve as the ion-buffering reservoirs, mesopores (2–50 nm) serve as the electrolyte ions transport channels, and micropores (<2 nm) usually serve as the charge storage sites [9,10,11]. Recently, various efforts have been made to synthesize the HPAC [5,6,7,8,9,10,11,12,13,14,15,16,17]. Given by such distinctive contributions of HPAC, their electrochemical performances including specific capacitance, rate capabilities, cycle stability, etc. were enhanced accordingly.

Except for the development of active materials, it is known that the technique adopted for electrode preparation would be one of the critical aspects, which significantly affected the corresponding electrochemical performances. Currently, most of the electrodes are made through the slurry-casting procedures. However, these processes could lead to the following issues: (1) capital expense, (2) energy consumption for removing solvents, and (3) performance fading caused by the solvent residues in the electrode [18]. Additionally, it is not easy to prepare the slurry-casted electrode from the active materials with very high surface area (e.g., graphene, HPAC, etc.), since excessive solvents are required for homogeneously dispersing them and conductive agents. Hence, the resulting electrode possessed a low-mass loading and exhibited insufficient energy density. To help fill the gaps between new materials and practical applications, Dong et al. summarized the recent design and preparation of high-mass loading carbon electrodes for SCs from the aspects of entire conductive networks and ion-transport channels, dense structure design, and freestanding electrode construction [19]. Among the strategies mentioned above, simultaneously maintaining their high electronic conductivity and ion-diffusion ability within the high-mass loading carbon electrodes remains challenging. Consequently, developing an efficient and environment-friendly technique to successfully prepare the HPAC-based electrode with high mass-loading, fast charge transfer, and ion migration is emergently desirable.

In this study, the alternative procedures including mechanical blending, calendering, and heating process were adopted to prepare the high-mass loading HPAC electrode as compared with the slurry-casted one. The morphology, water repellency, mechanical, and electronic properties of the HPAC electrode were sequentially characterized. The capacitive performance, rate capability, and cycling stability were systematically evaluated using cyclic voltammetry and galvanostatic charge–discharge methods in the presence of 1 M tetraethylammonium tetrafluoroborate/propylene carbonate (TEABF_4_/PC) organic electrolyte under the symmetric coin and pouch-type cells. To the best of our knowledge, this is the first demonstration of utilizing the HPAC as starting materials to produce the electrode with high-mass loading by the alternative processes for pouch-type SCs. This approach may also offer new possibilities to prepare a variety of high-mass loading electrode using different active materials for extensive applications.

## 2. Materials and Methods

### 2.1. Preparation of High-Mass Loading Hierarchically Porous Activated Carbon (HPAC) Electrode

The high-mass loading hierarchical porous activated carbon (HPAC) electrode is composed of HPAC, carbon black (Super P^®^, Timcal Ltd., Bodio, Switzerland), and colloidal polytetrafluoroethylene (PTFE) dispersion (D1-E, Daikin Industries Ltd., Osaka, Japan). Synthetic procedures and the relative characterization of the HPAC used in this study were reported in our previous study [20]. Briefly, a pyrolysis process was firstly utilized to transform rubberwood sawdust into bio-oil under an N_2_ atmosphere at 500 °C through a fluidized-bed reactor. After that, the HPAC was obtained by carbonizing the bio-oil/ZnO nanoparticles precursor mixture (weight ratio of 1:4) at 900 °C under an N_2_ atmosphere for 4 h and washing with HCl solution, deionized (DI) water, and drying in an oven. Particularly, the specific surface area of the as-synthesized HPAC was 1365 m^2^/g, with a microporous area of 530 m^2^/g and mesoporous/external area of 835 m^2^/g. To prepare the HPAC electrode, the ingredients (82 wt % of HPAC, 10 wt % of Super P^®^, and 8 wt % of D1-E) were mechanically blended by a planetary mixer (PM 200, Retsch GmbH, Haan, Germany). The resulting mixture was repeatedly calendered using a rolling presser (MSK-2150-DC, MTI corporation) under the ambient condition. The as-prepared HPAC electrode with a thickness of 230 ± 10 µm was obtained after it was heated at 130 °C for 2 h. The mass-loading of HPAC is 8.3 ± 0.2 mg/cm^2^, which is about 3.6 times higher than that of the slurry-casted electrode [20].

### 2.2. Characterizations

For morphological observations, the top-view and cross-sectional micrographs were acquired using a field-emission scanning electron microscope (FE-SEM, S-4200, Hitachi, Ltd., Tokyo, Japan) operated at 15 kV. A contact angle test was measured by a video contact angle instrument (OCA 15 plus, Dataphysics Instruments GmbH, Filderstadt, Germany). The mechanical and electronic properties were determined using a high-precision tensile tester equipped with a 500 N load cell (EZ-SX short model, Shimadzu Corp., Kyoto, Japan) and four-point probe analyzer (NAP-RT-70/RG-7B, Napson Corp., Tokyo, Japan), respectively. The sample used for mechanical property was prepared according to ASTM D638.

### 2.3. Electrochemical Measurements

The electrochemical tests throughout this study were conducted in a symmetric two-electrode configuration at ambient condition, and the voltage window was in the range of 1.35 and 2.7 V. Prior to fabricating the testing cell, the HPAC electrode was attached onto a carbon-coated aluminum foil by a rolling presser. The working areas used for a coin (CR 2032) and pouch-type cell were 1.33 cm^2^ and 24.5 cm^2^, respectively. The organic electrolyte of 1 M TEABF_4_/PC and cellulose-based separator (TF4535, NKK, Kanagawa, Japan) were chosen. With regard to the assembly of the pouch cell, it was vacuum-sealed after the electrolyte was completely absorbed into the electrodes via a vacuum-assisted process. The cyclic voltammograms (CV) and rate capabilities of coin cells were recorded using a multichannel electrochemical workstation (VMP3, Bio-Logic, Seyssinet-Pariset, France). The scan rates used for the former were from 1 to 10 mV/s, whereas the current density applied for the latter were from 1 mA/cm^2^ (0.12 A/g) to 10 mA/cm^2^ (1.2 A/g). The cycling stability of the coin cell was measured at the current of 5 mA (i.e., 0.45 A/g and 3.76 mA/cm^2^). The rate capabilities and cycling stability of the pouch-type supercapacitor (1 cell) were galvanostatically tested. The current densities used in rate capabilities were the same as those for the coin cells, but we applied a current of 245 mA (i.e., 1.2 A/g and 10 mA/cm^2^) to evaluate the cycling stability. The pouch cell assembled by integrating 10 pieces of electrodes (i.e., 5 cells, Figure S1) further measured the cycling stability at a current of 1 A through a computer-controlled system (Series 4000, Maccor, Inc., Tulsa, OK, USA) to evaluate its practical application of such HPAC electrode. The values of energy (Wh) and power (W) can be directly obtained through EC-lab^®^ software. Then, the energy and power densities were calculated by dividing the total weight of active materials at the two electrodes.

## 3. Results and Discussion

### 3.1. Morphologies and Properties of High-Mass Loading Hierarchically Porous Activated Carbon (HPAC) Electrode

**Figure 1:**
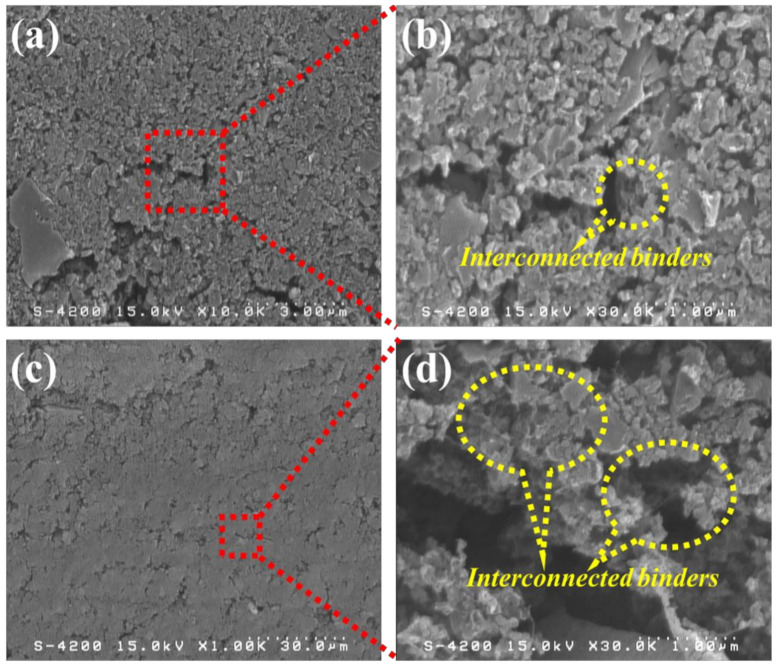
SEM micrographs of the high-mass loading hierarchically porous activated carbon electrode: (**a**,**b**) top-view and (**c**,**d**) cross-section. Scale bars of (**a**–**d**) are 3, 30, and 1 μm, respectively.

Figure 1 shows the top-view and cross-sectional micrographs of the high-mass loading hierarchically porous activated carbon (HPAC) electrode revealed by FE-SEM. As can be seen, the smooth morphology with microporous structure was revealed in Figure 1a, which was quite similar with that of PTFE/carbon black composites [21]. Notably, numerous voids remain within the HPAC electrode (Figure 1a,c) even it was repeatedly calendered during preparation. Such microstructures would be beneficial for electrolyte penetration throughout the HPAC electrode and further well-absorbed into the HPAC particles. In addition, the PTFE fibers bound on the HPAC and interconnected in the matrix are clearly observed (highlighted in Figure 1b,d). It is anticipated that the interconnected binders not only maintained the mechanical strength of the HPAC electrode but also contributed significant water repellency to avoid any moisture absorbed by the hygroscopic HPAC particles.

**Figure 2:**
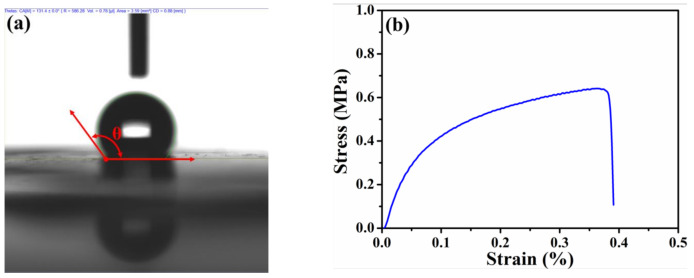
The deionized (DI) water contact angle (**a**) and stress–strain curve (**b**) of the high-mass loading hierarchically porous activated carbon electrode.

To understand the water-repellency, mechanical, and electronic properties of the HPAC electrode, the corresponding measurements were conducted, while their results are discussed as follows. Owing to the presence of oxygen-containing function groups as evidenced in the previous study [20], the hygroscopicity of the HPAC is obviously revealed (Figure S2). It has been reported that such function groups can lead to additional active sites in enhancing the capacitances [22,23]. However, the moisture residues that remain within the HPAC would cause a negative effect on electrochemical performances, especially for an organic electrolyte. Interestingly, the average contact angle of the HPAC electrode is 133.5 ± 2° (representative DI water contact angle of the HPAC electrode was shown in Figure 2a), indicating the significant hydrophobicity. The flexibility of the high-mass loading electrode is an imperative property so that it can avoid any undesired crack within the electrode and facilitate continuous electron transportations. Figure S3 shows the digital image of the HPAC electrode after winding. Obviously, the smooth surface of the HPAC electrode without any destruction was found, demonstrating its flexibility. On the other hand, the tensile strength has also been seriously considered. The representative stress–strain curve of the HPAC electrode is displayed in Figure 2b. As a result, the yield strength and yield elongation are 0.6 ± 0.11 MPa and 0.3 ± 0.05%, respectively. The less elongation is attributable to a decrease of the PTFE fibers formed from the colloidal PTFE dispersion interconnecting throughout the matrix [21,24]. Even that, the HPAC electrode is tough enough for assembling the testing cells. The electronic property of high-mass loading electrode is another critical feature especially for high-power applications because the electrons are necessary to transport fast. The average sheet resistance of the HPAC electrode measured by a four-point probe analyzer is 23 ± 1.7 Ω/sq. According to the results discussed above, it is rationally anticipated that the HPAC electrode thus prepared could exhibit good electrochemical performances for SCs.

### 3.2. Electrochemical Performances of High-Mass Loading Hierarchically Porous Activated Carbon (HPAC) Electrode

**Figure 3:**
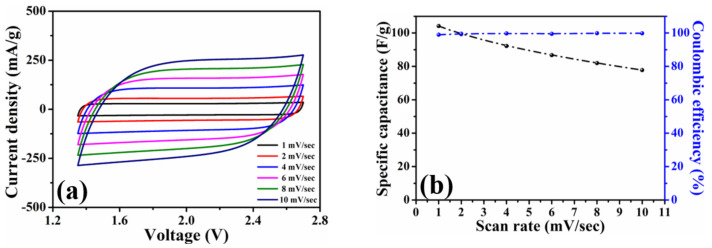
(**a**) Cyclic voltammograms and (**b**) average specific capacitances of a coin-type symmetric cell with high-mass loading hierarchically porous activated carbon electrodes recorded in 1.35–2.7 V at scanning rates from 1 to 10 mV/s.

To investigate the capacitive efficiencies of the HPAC electrode, the coin-type symmetric cell was first assembled to conduct the cyclic voltammetry and galvanostatic charge–discharge measurements at different scanning rates and current densities. Figure S4 shows the cyclic voltammogram of the HPAC electrode recorded in the voltage range of 0 to 2.7 V at a scanning rate of 1 mV/s. Benefiting from the high specific surface area and hierarchical porous frameworks, the conventional rectangular contour was revealed, although the mass loading and thickness were much higher than the electrode that was prepared by doctor-blade method [13,20]. The specific capacitance (C, F/g) over the whole voltage range was calculated to be 490 F/g (2.17 C/cm^2^) from the cyclic voltammogram by C = 4 *I*dt/*MV*, where *I* is discharge current, t is the discharge time, *V* is the voltage window, and *M* is the total weight of active materials at the two electrodes [7]. To carry out the accelerated experiments, the voltage range from 1.35 to 2.7 V (i.e., 50% of the state of discharge) was further utilized. As can be seen, the shape of each cyclic voltammogram in Figure 3a kept the nearly rectangular curve with good symmetry and was not significantly affected after gradually increasing the scanning rates. The specific capacitances compared in Figure 3b reveal that the value obtained at 10 mV/sec is 77.8 F/g, which was about 75% of the value recorded from 1 mV/sec. Those results reflect that >99% of Coulombic efficiency and high-rate capability of the HPAC were achieved even in such high-mass loading and a thick electrode.

**Figure 4:**
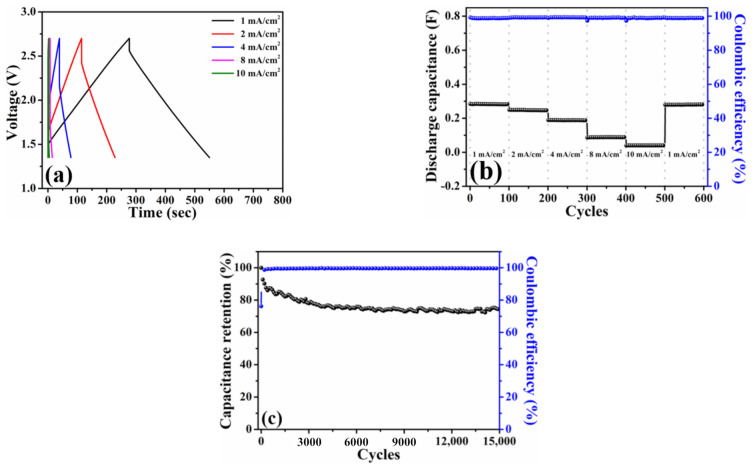
(**a**) Galvanostatic charge–discharge profiles, (**b**) rate capabilities, and (**c**) capacitance retention of a coin-type symmetric cell with high-mass loading hierarchically porous activated carbon electrodes in 1.35–2.7 V. The current densities used in (**a**,**b**) are 1 mA/cm^2^ (0.12 A/g) to 10 mA/cm^2^ (1.2 A/g), whereas the current used in (**c**) is 5 mA (i.e., 0.45 A/g and 3.76 mA/cm^2^).

Figure 4a plots the galvanostatic charge–discharge profiles of the HPAC electrode measured at current densities from 1 mA/cm^2^ (0.12 A/g) to 10 mA/cm^2^ (1.2 A/g). Thanks to the ideal electric double-layer behavior as presented in Figure 3a, a symmetric charge–discharge curve collected at each current density was exhibited. The discharge capacitance delivered from this coin-type symmetric cell is about 0.28 F at 1 mA/cm^2^ with the Coulombic efficiency of 99%. In addition, the stable discharge capacitances of 0.25, 0.19, 0.09, and 0.04 F at 2, 4, 8, and 10 mA/cm^2^ are compared in Figure 4b. Notably, almost full recovery of the initial capacity and no capacity fading after 100 cycles were achieved when the current density was returned to 1 mA/cm^2^. The cycling stability was further measured at the current of 5 mA (i.e., 0.45 A/g and 3.76 mA/cm^2^), whereas the corresponding result is shown in Figure 4c. As indicated, approximate 74% capacitance retention and ≥99.5% Coulombic efficiency were delivered after 15,000 cycles, respectively. These positive findings that are reflected from the electrochemical measurements of a coin-type symmetric cell encourage us to explore the practical applicability of the HPAC electrode by the pouch-type symmetric device.

**Figure 5:**
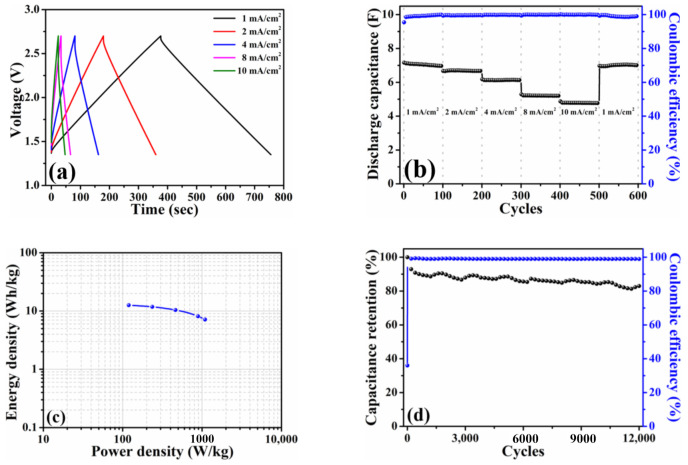
(**a**) Galvanostatic charge–discharge profiles, (**b**) rate capabilities, (**c**) Ragone plot, and (**d**) capacitance retention of a pouch-type symmetric cell with high-mass loading hierarchically porous activated carbon electrodes in 1.35–2.7 V. The current densities used in (**a**,**b**) are 1 mA/cm^2^ (0.12 A/g) to 10 mA/cm^2^ (1.2 A/g), while the current used in (**c**) is 245 mA (1.2 A/g and 10 mA/cm^2^).

The pouch-type symmetric device (1 cell) was fabricated to evaluate the electrochemical performances under the conditions same as Figure 4. Similarly, the galvanostatic charge–discharge profiles depicted in Figure 5a exhibited a symmetric charge–discharge curve at each current density same as the trends observed in Figure 4a. It is apparently distinguished that the voltage drops from the pouch-type symmetric cell were slight when compared with those of coin-type counterpart. Generally, the voltage drop is resulted from electrolyte potential drop and contact resistance, and it is also dependent on the charge–discharge current density [25]. The electrolyte and current density used in the present study were kept constant while the pouch cell was assembled by vacuum sealing. Hence, it could attribute the difference in voltage drop between the coin cell and pouch cell to the contact resistance. Even in the larger working area (24.5 cm^2^), the stable discharge capacitances (7.1 F at 1 mA/cm^2^ (0.12 A/g) and 4.8 F at 10 mA/cm^2^ (1.2 A/g)) and Coulombic efficiencies (≥99% for all current densities) were noticed in Figure 5b. The Ragone plot calculated based on the total mass of the HPAC is illustrated in Figure 5c. The energy density was in the range of 12.6 to 7.1 Wh/kg with the power density of 119.4 to 1090.1 W/kg. To explore the influence of voltage range on the energy and power densities of the HPAC electrode, the pouch-type symmetric cell was galvanostatic tested between 0 and 2.7 V. It is revealed that the charge–discharge profile (Figure S5a) obtained at each current density was the same as those depicted in Figure 5a. Interestingly, the energy density was increased from 12.6 to 17.5 Wh/kg when the cell was subjected to 100% of the state of discharge. However, its power density was shifted to 661.7 W/kg (Figure S5b). As reported in [19], increasing the mass loading or electrode thickness results in the fading of the capacitance and rate performance of the electrode materials because of the decreased accessible surface area, enlarged electrical resistance, prolonged ion transport channels, and poor electrolyte wetting. Among those factors mentioned above, the lower power density outputted from the HPAC electrode might be correlated to electrical resistance even only 8 wt % of the insulting PTFE binder was used. To further enhance the rate performance, creating the extra electron transport pathways through the addition of one-dimensional carbon materials would be helpful. After 12,000 cycles at the current of 245 mA (1.2 A/g and 10 mA/cm^2^), the capacitance retention and Coulombic efficiency shown in Figure 5d were about 83% and 99%, respectively. Since the pouch-type symmetric device was vacuum-sealed after the electrolyte was completely absorbed into the HPAC electrodes via a vacuum-assisted process, it is reasonably expected that air bubbles within the HPAC electrode were entirely removed. Consequently, it would avoid any side reaction occurring during the measurement, resulting in better capacitance retention in comparison with that obtained from the coin-type symmetric cell (Figure 4c). Those resulting capacitive performances could be contributed by the fascinating properties of HPAC and the synergistic features (i.e., microporous structures, significant hydrophobicity, and sufficient mechanical and electronic properties) of the HPAC electrode.

**Figure 6:**
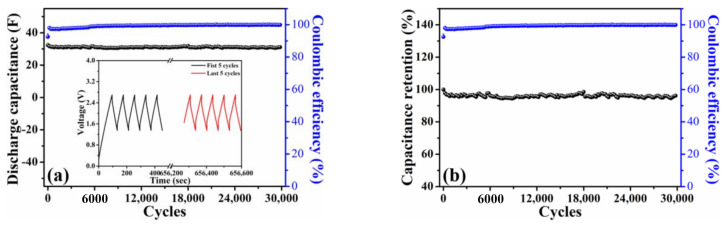
(**a**) Discharge capacitance as a function of cycle number and (**b**) capacitance retention of a pouch-type symmetric cell integrating by 10 pieces of high-mass loading hierarchically porous activated carbon electrodes in 1.35–2.7 V. The current used in is 1 A. Inset of (**a**) illustrates the galvanostatic charge–discharge profiles of the first and last five cycles.

Another pouch-type symmetric device achieved by integrating 10 pieces of the HPAC electrodes (i.e., 5 cells, Figure S1) was further assembled to examine the cycling stability at a current of 1 A. Figure 6a shows that such a device was able to deliver an initial discharge capacitance of 32.4 F with the Coulombic efficiency of 93%. Their galvanostatic charge–discharge profiles of the first and last five cycles in the inset of Figure 6a presented the symmetric shape, revealing the highly reversibility even after 30,000 cycles. It is worth mentioning that 96% of capacitance retention (≈0.013% fading per cycle) and ≥99% of Coulombic efficiency was achieved (Figure 6b). To the best of our knowledge, this is the first report using the HPAC powders as starting materials to successfully prepare the high-mass loading electrode as compared with the slurry-casted one by the alternative processes. Moreover, the stable cycling performances for the SCs in an organic electrolyte system were demonstrated, which could have potential for the practical applicability.

## 4. Conclusions

In summary, this study presents the alternative processes to successfully prepare a high-mass loading electrode using the hierarchical porous activated carbon (HPAC) as starting materials. The resulting HPAC electrode exhibits the smooth and microporous morphology, significant hydrophobicity, and sufficient mechanical and electronic properties as evidenced by the corresponding characterizations. Thanks to the fascinating properties of the HPAC particles and the synergistic features of the HPAC electrode, the pouch-type symmetric device using the propylene carbonate-based electrolyte delivered the favorable electrochemical properties including capacitive performance (7.1 F at 1 mA/cm^2^), Coulombic efficiency (≥99%), rate capability (4.8 F at 10 mA/cm^2^), and capacitance retention (83% at 12,000 cycles). Encouragingly, an alternative pouch-type symmetric device (i.e., 5 cells) not only presented an initial discharge capacitance of 32.4 F but also achieved 96% of capacitance retention (≈0.013% fading per cycle) and ≥99% of Coulombic efficiency after 30,000 cycles. However, it is revealed that the energy and power densities outputted from the HPAC electrode were not competitive with previous reports. For further improvements, incorporating the one-dimension conductive materials (e.g., carbon nanotubes/fibers) to construct the extra electron transport pathways and optimizing the corresponding composition would be beneficial for realizing the practical applicability of the HPAC electrode prepared in the present study.

## Data Availability

The data is available on reasonable request from the corresponding author.

## Supplementary Materials

The following are available online at https://www.mdpi.com/2079-4991/11/3/785/s1, Figure S1: Digital photograph of the pouch-type symmetric device (5 cells), Figure S2: DI water contact angle test on the HPAC flake, Figure S3: Digital image of the HPAC electrode after winding, Figure S4: Cyclic voltammogram of the HPAC electrode recorded in the voltage range of 0 to 2.7 V at a scanning rate of 1 mV/s, Figure S5: (a) Galvanostatic charge–discharge profiles and (b) Ragone plot of a pouch-type symmetric cell with high-mass loading hierarchically porous activated carbon electrodes measured in the voltage range of 0 to 2.7 V.
