# Supplementary material for: High-Mass Loading Hierarchically Porous Activated Carbon Electrode for Pouch-Type Supercapacitors with Propylene Carbonate-Based Electrolyte
Source: Nanomaterials (Basel). 2021 Mar 19;11(3):785. doi: 10.3390/nano11030785 (PMC8003487; doi:10.3390/nano11030785)
Supplement: Supplementary file 1 [file nanomaterials-11-00785-s001.pdf]

**Supporting Information**

# High-mass loading hierarchically porous activated carbon electrode for pouch-type supercapacitors with propylene carbonate-based electrolyte

Tai-Feng Hung [1,*], Tzu-Hsien Hsieh [2], Feng-Shun Tseng [3], Lu-Yu Wang [3],

Chang-Chung Yang [3], Chun-Chen Yang [1,4,5,*]

[1]Battery Research Center of Green Energy, Ming Chi University of Technology, 84 Gungjuan Rd., Taishan Dist., New Taipei City 24301, Taiwan

[2]Green Technology Research Institute, CPC Corporation, Taiwan, 2 Zuonan Rd., Nan-Tsu Dist., Kaohsiung 81126, Taiwan

[3]Energy Storage Technology Division, Green Energy & Environment Research Laboratories, Industrial Technology Research Institute, 301 Gaofa 3rd Rd., Guiren Dist., Tainan 71150, Taiwan

[4]Department of Chemical Engineering, Ming Chi University of Technology, 84 Gungjuan Rd., Taishan Dist., New Taipei City 24301, Taiwan

[5]Department of Chemical and Materials Engineering, Chang Gung University, 259 Wenhua 1st Rd., Guishan Dist., Taoyuan 33302, Taiwan

[*] Corresponding author.

*E-mail addresses*: taifeng@mail.mcut.edu.tw (T.F. Hung)

ccyang@mail.mcut.edu.tw (C.C. Yang)

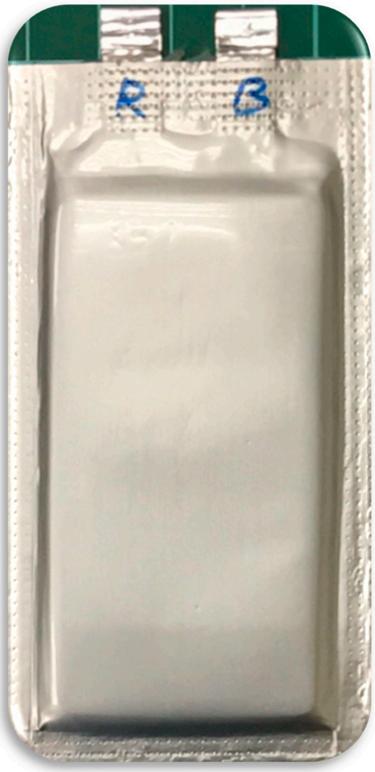


**Figure S1.** Digital photograph of the pouch-type symmetric device (5 cells), electrode area: 3.5 cm* 7 cm (W*L).

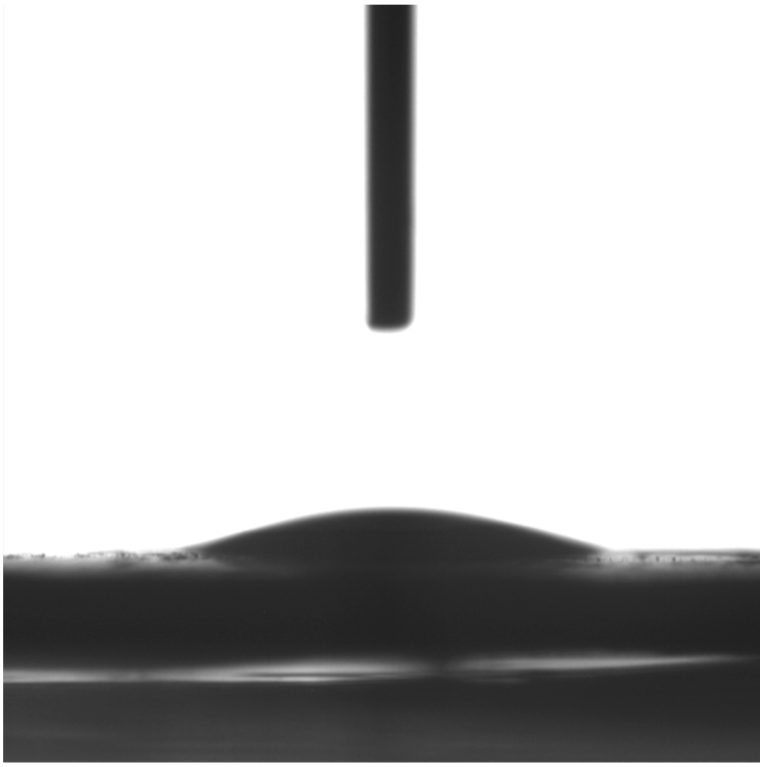

**Figure S2.** DI water contact angle test on the HPAC flake.

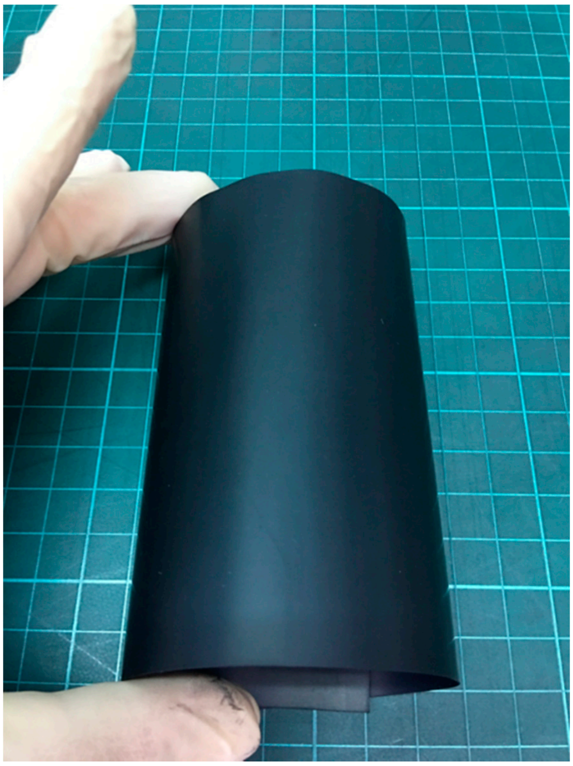

**Figure S3.** Digital image of the HPAC electrode after winding.

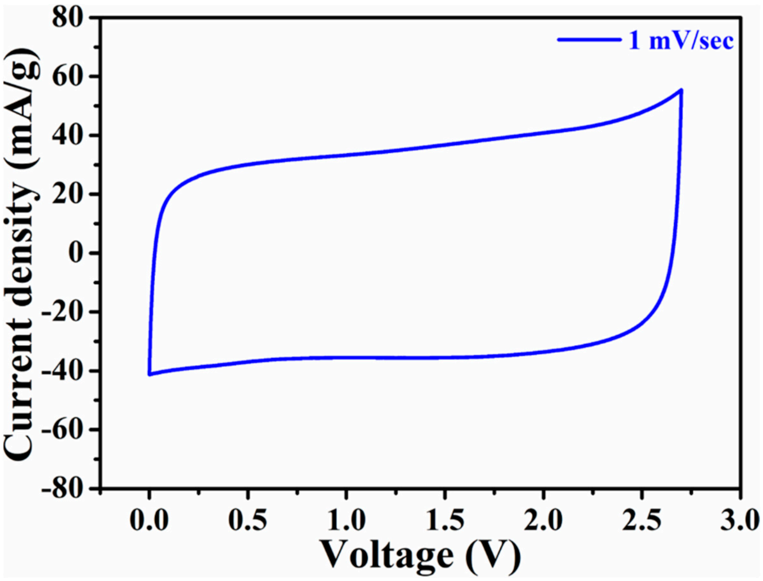


**Figure S4.** Cyclic voltammogram of the HPAC electrode recorded in the voltage range of 0 to 2.7 V at a scanning rate of 1 mV/sec.

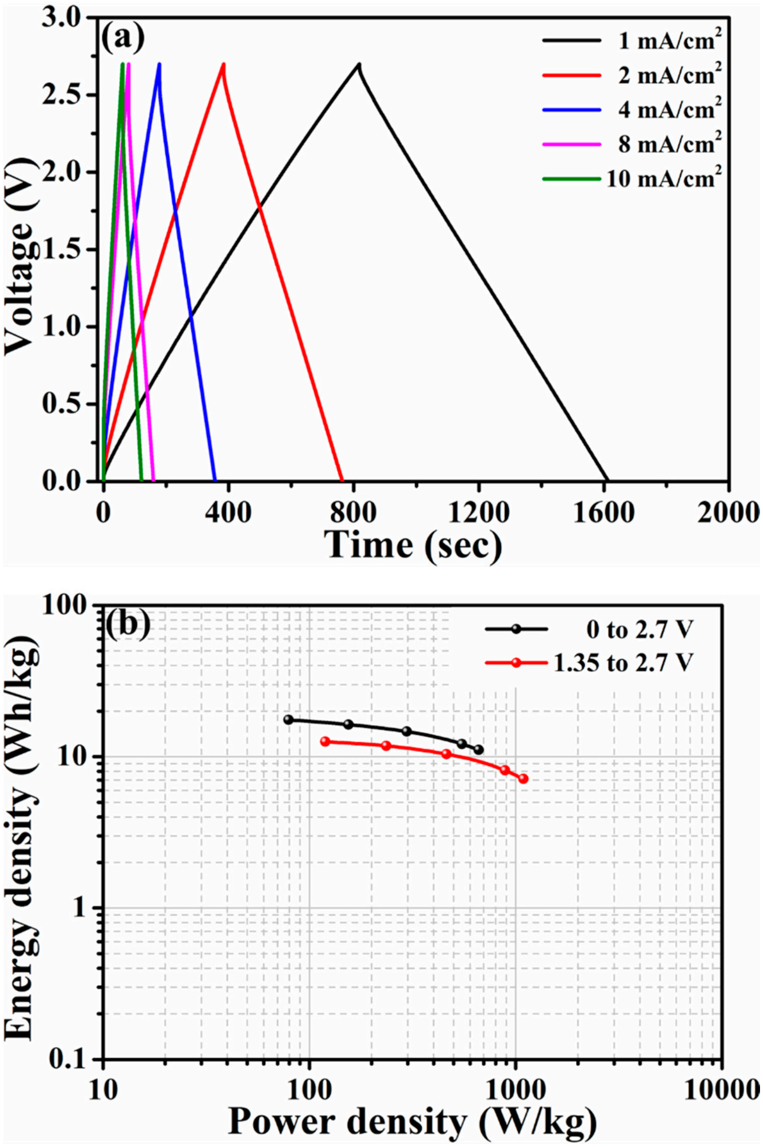


**Figure S5.** (a) Galvanostatic charge-discharge profiles and (b) Ragone plot of a pouch-type symmetric cell with high-mass loading hierarchically porous activated carbon electrodes measured in the voltage range of 0 to 2.7 V. The current densities used in (a) are 1 mA/cm$^2$ (0.12 A/g) to 10 mA/cm$^2$ (1.2 A/g).
